# The Combination of Immune Checkpoint Blockade with Tumor Vessel Normalization as a Promising Therapeutic Strategy for Breast Cancer: An Overview of Preclinical and Clinical Studies

**DOI:** 10.3390/ijms24043226

**Published:** 2023-02-06

**Authors:** Ombretta Melaiu, Gianluca Vanni, Ilaria Portarena, Chiara Adriana Pistolese, Lucia Anemona, Silvia Pomella, Roberto Bei, Oreste Claudio Buonomo, Mario Roselli, Alessandro Mauriello, Giovanni Barillari

**Affiliations:** 1 Department of Clinical Sciences and Translational Medicine, University of Rome Tor Vergata, Via Montpellier 1, 00133 Rome, Italy; 2 Breast Unit, Department of Surgical Science, PTV Policlinico “Tor Vergata” University, Via Montpellier 1, 00133 Rome, Italy; 3 Medical Oncology Unit, Department of Systems Medicine, University of Rome Tor Vergata, Via Montpellier 1, 00133 Rome, Italy; 4 Department of Diagnostic and Molecular Imaging, Interventional Radiology and Radiotherapy, University Hospital of Rome “Tor Vergata”, Viale Oxford 81, 00133 Rome, Italy; 5 Anatomic Pathology, Department of Biomedicine and Prevention, University of Rome Tor Vergata, Via Montpellier 1, 00133 Rome, Italy

**Keywords:** breast cancer, angiogenesis, vessel normalization, tumor microenvironment, immunosuppression, antitumor immunity, immune checkpoint inhibitors

## Abstract

Immune checkpoint inhibitors (ICIs) have a modest clinical activity when administered as monotherapy against breast cancer (BC), the most common malignancy in women. Novel combinatorial strategies are currently being investigated to overcome resistance to ICIs and promote antitumor immune responses in a greater proportion of BC patients. Recent studies have shown that the BC abnormal vasculature is associated with immune suppression in patients, and hampers both drug delivery and immune effector cell trafficking to tumor nests. Thus, strategies directed at normalizing (i.e., at remodeling and stabilizing) the immature, abnormal tumor vessels are receiving much attention. In particular, the combination of ICIs with tumor vessel normalizing agents is thought to hold great promise for the treatment of BC patients. Indeed, a compelling body of evidence indicates that the addition of low doses of antiangiogenic drugs to ICIs substantially improves antitumor immunity. In this review, we outline the impact that the reciprocal interactions occurring between tumor angiogenesis and immune cells have on the immune evasion and clinical progression of BC. In addition, we overview preclinical and clinical studies that are presently evaluating the therapeutic effectiveness of combining ICIs with antiangiogenic drugs in BC patients.

## 1. Introduction

The advent of immunotherapy has paved the way for treating highly aggressive, previously incurable cancers in a considerable percentage of patients [1]. In particular, immune checkpoint (IC) inhibitors (ICIs) that reactivate dysfunctional and/or exhausted T cells have shown remarkable efficacy against a wide range of solid and hematologic tumors [2]. However, the administration of ICIs as monotherapy has shown limited efficacy and high side effects in certain types of tumors [3]. Among the latter is breast cancer (BC) [4,5,6], the most common female malignancy worldwide [7], which, being poorly immunogenic, has for a long time been considered as generically resistant to immunotherapy [8]. In the last two decades, however, it has been understood that this concept does not apply indiscriminately to all BC patients [9]. In fact, the thorough characterization of BC heterogeneity has allowed the delineation of molecular subtypes with specific pathological features and clinical outcomes [9]. BC subtypes include luminal A, luminal B, human epidermal growth factor receptor 2 (HER2)-positive, and basal-like BC [10]. Luminal tumors express hormone receptors [11], while HER2-positive tumors are characterized by HER2 overexpression [11]. On their part, basal-like BCs express neither hormone receptors nor HER2, thereby being named triple-negative BCs (TNBCs) [11]. Amidst the BC subtypes, TNBC has emerged as an attractive candidate for the evaluation of novel immunotherapy approaches. This is because TNBC displays high genomic instability that leads to the generation of tumor-specific neoantigens, overexpression of the IC programmed death-ligand 1 (PD-L1), and a high density of tumor-infiltrating lymphocytes (TILs) [12,13,14]. Altogether, these features argue in favor of TNBC responsiveness to immunotherapy. Therefore, in an attempt to overcome the modest clinical activity of ICIs administered as monotherapy, treatments involving the combination of ICIs and immunogenic chemotherapy are being evaluated [4,6].

To date, two immunotherapy agents, atezolizumab (an antibody directed against PD-L1) and pembrolizumab (an anti-PD1 antibody), have been approved in combination with chemotherapy for PD-L1 positive, advanced TNBC [15,16]. Interestingly, patients who have received cisplatin or doxorubicin followed by the administration of nivolumab (another antibody against PD1) experience the upregulation of immune-related genes involved in PD-1/PD-L1 and cytotoxic T-cell pathways (NCT04159818 [17]). Several studies have also evaluated ICIs’ combination with chemotherapeutics during neoadjuvant treatment [18]. However, the addition of PD-1/PD-L1 inhibitors to chemotherapy has been reported to increase the rate of pathologic complete response (pCR) only in a small fraction of patients with early-stage BC [6]. Indeed, most patients respond initially and then develop resistance [6]. In addition, unsatisfactory activity has been observed in BC subtypes other than PD-L1-positive TNBCs [6].

To date, no solid data can accurately explain the pattern of response or resistance to ICIs in BC patients [19]. Thus, a deeper understanding of the mechanisms for BC resistance to immunotherapy is required to achieve pCR in a greater number of patients. In this regard, the analysis of the tumor microenvironment (TME) holds great promise [19].

The TME consists of stromal and immune cells lying on an extracellular matrix traversed by blood and lymphatic vessels [20]. Compared with its non-malignant counterpart, each component of the TME is abnormal in a fashion that fuels tumor progression and resistance to therapy [21]. In particular, the TME of BC is characterized by hypoxia, a low pH, and a high interstitial fluid pressure [22,23]: all these features not only reduce the efficacy of anticancer therapies, but also hamper immune cells entrance in the tumor nest [24]. This evidence suggests that normalizing the TME of BC could improve the efficacy of antitumor chemo/immunotherapy.

In this review, we focus on one important component of the TME, the tumor vessels. Specifically, we discuss the strategies currently under investigation to improve the effectiveness of immunotherapy via the normalization of BC vasculature.

## 2. The Abnormal Tumor Vasculature of Breast Cancer

In tumors, the formation of new blood vessels is mainly accomplished through angiogenesis, the multistep process in which endothelial cells lining pre-existing vessels degrade the basement membrane and migrate into the perivascular space to form capillary structures: the latter will eventually cavitate, permitting blood influx [25,26,27]. Tumor vessel formation is boosted when angiogenesis is accompanied by vasculogenesis, a process involving the recruitment of immature endothelial cell precursors from the bone marrow to the nascent vessels [28]. Additional events leading to the expansion of the tumor vasculature are the vasculogenic mimicry (in which cancer cells form channels allowing blood inflow), and the vascular co-option (in which cancer cells line the abluminal surface of pre-existing normal vessels) [29].

Whatever the mechanism is that is responsible for their development, the newly formed blood vessels display both protumor and antitumor properties: in fact, if on the one hand they supply oxygen and nutrients to the neoplastic mass and allow the spread of metastatic cells, on the other hand they favor the infiltration of the tumor by the immune cells [30].

Notably, unlike normal vessels, tumor vessels are tortuous and chaotically organized, with wide gaps between endothelial cells, detached pericytes, and basal membranes that are either too thick or too thin [21]. All these features are typical of BC [31]. Importantly, BC biopsies are routinely evaluated for vessel density, which reliably predicts the risk of BC recurrence/metastasis and, thereby, BC patients’ survival rate [32,33].

Of upmost interest, a previous in vivo study showed that inoculating BC cells at different anatomical sites (mammary gland, cranium, and dorsal skin) leads to the establishment of abnormal vascularization whose features significantly differ from site to site [34].

However, whatever the involved anatomic site is, tumor vessels are characterized by a loss of structural integrity and functional aberrations that promote inflammation and tissue fibrosis, and, at the cellular level, DNA hypermethylation, genomic instability, trans-differentiation, and resistance to apoptosis [35]. The proliferating tumor mass squeezes blood vessels leading to flow stasis and thereby limiting the access of both drugs and immune cells to the tumor [36]. In addition, flow stasis causes vessel permeability and blood concentration, thus lowering tissue pH and oxygen [36]. Notably, due to their abnormal vasculature, about 25–40% of invasive BC exhibits hypoxic regions [37,38]. There, the hypoxia-inducible transcription factor (HIF) is activated, leading to the expression of molecular players that further stimulate angiogenesis [39]. These reciprocal interactions establish a vicious cycle [39] that contributes to BC aggressiveness and/or its resistance to therapy [38]. Concerning this last aspect, it is well established that the abnormal structure of tumor vessels can hamper the homogeneous distribution of anticancer drugs within the tumor [40,41]. In addition, one should consider that to be fully effective, chemotherapeutics, such as those currently employed to treat BCs, require adequate oxygen levels [35,42]. Moreover, because of the prevalence of leaky vessels in the tumor, tumor cells enter the systemic circulation, eventually giving rise to metastases [43]. The latter are the major cause of morbidity and mortality among BC patients [44]. Approximately 20–30% of early-stage BC patients will develop metastases, most frequently at the liver, lung, bone, or brain [44]. Despite therapeutic advances in BC, prevention of metastasis is still a challenge, and aberrant angiogenesis is the essential early stage of this complex process [45].

Finally, the hypervascularization of BC regulates the dysfunctional homing of lymphocytes. These events lead to immunosuppression, reduced immune surveillance, and poor trafficking of immune effector cells to the TME [46,47]. It is therefore reasonable to speculate that the abnormal vasculature could favor BC resistance to immunotherapy by jeopardizing the adhesion of immune effector cells to the endothelium and their intrusion into TME.

### The Molecular Players of Aberrant Vasculature in Breast Cancer

Dysregulated tumor-associated angiogenesis is orchestrated by a variety of molecular players, such as vascular endothelial growth factor (VEGF), interleukin 8 (IL-8), pleiotrophin, angiopoietin-1, angiopoietin-2, platelet-derived growth factor (PDGF), fibroblast growth factor (FGF)-2, and transforming growth factor beta-1 (TGFβ-1). Clinical studies have shown that elevated levels of these cytokines are associated with a worse prognosis of several tumor types [48,49,50] and also play a critical role in BC progression [31,51].

The VEGF family (VEGF-A–F and their receptors, VEGFR-1–3 and neuropilin) are key to the angiogenesis associated to BC [52,53,54,55]. In addition, the binding of VEGF-A to VEGFR-1 or VEGFR-2 has a role in BC development [56,57], while VEGF-D is important to BC metastasization via lymphatic vessels [55]. Consistently, VEGF levels in tumor tissue or serum positively correlate with the severity of the prognosis of BC patients [53,58,59].

Others have shown that BCs expressing high levels of IL-8 are particularly aggressive and invasive [60]. In fact, IL-8 triggers the expression of the extracellular-matrix-degrading matrix metalloproteinases (MMPs) enzymes, which, in turn, promote both tumor cell invasion and angiogenesis [61]. Notably, IL-8-overexpressing BC cell lines also synthesize high levels of VEGF [60,62,63,64], whose pro-tumorigenic activities are potentiated by IL-8 [61,65]. As for VEGF, the FGFs also spark angiogenesis in BC. In this context, it is of note that the expression of FGF-2 is increased in patients treated with VEGF antagonists [66]. For its part, angiopoietin 2, which is predominantly found in hypoxic tumor tissues [67,68], regulates the maturation of BC blood vessels by acting in a complementary manner to the VEGF pathway [69,70].

Additional factors that are expressed in BC tissues in a fashion that positively correlates with both the intensity of angiogenesis and tumor aggressiveness include TGFβ-1, pleiotrophin, placental growth factor, and PDGF [71].

In addition, the vasculogenic mimicry has been associated with poor prognosis, tumor aggressiveness, metastasis, and drug resistance in BC as well as other types of tumor [72,73].

## 3. Impact of Abnormal Breast Tumor Vascular on Immune Cells

**Figure 1:**
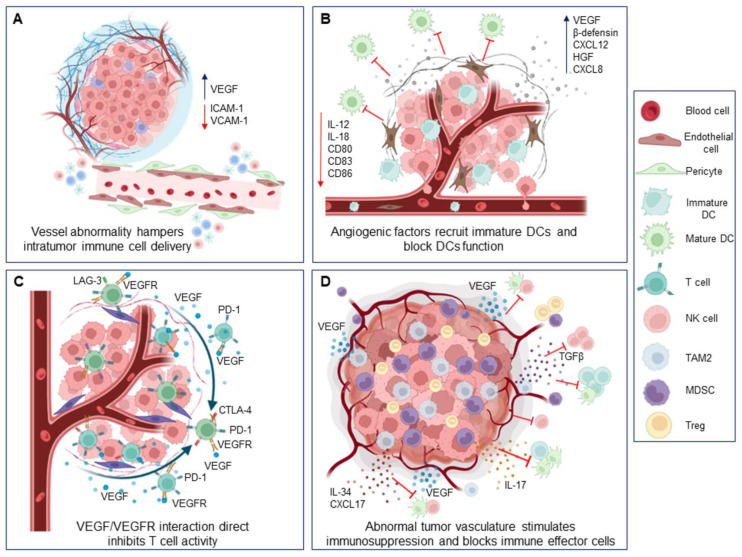
Abnormal tumor angiogenesis promotes tumor immune evasion. (**A**) Wide gaps between endothelial cells, detached pericytes, and thick/thin basement membranes, together with high levels of VEGF and low expression of endothelial adhesion molecules, reduce adhesion, extravasation, and infiltration of leukocytes into the tumor bed and contribute to establish the immune-desert and immune-excluded BC phenotype. (**B**) Ability of TME to promote the release of VEGF, other angiogenic factors, and protumor cytokines capable of inhibiting the maturation and function of DCs (characterized by low expression of CD80, CD83, CD86, etc.). (**C**) The exhaustion state of T cells is directly induced by the binding between the VEGF-R receptor and the VEGF-A ligand, major source of the inhibition of their effector function. (**D**) Vascular microenvironment produces multiple cytokines that recall immunosuppressive cells such as TAM2, Treg, and MDSC and reduce tumor infiltration and activity of DC, NK, and T cells. The figure was created with BioRender.com.

Antitumor immunity is exerted by both tissue-resident immune cells and those recruited intratumorally from the blood [74]. In this context, the abnormal tumor angiogenesis can be considered an important mechanism of immune evasion (Figure 1).

Indeed, an effective antitumor immune response requires not only the activation of effector immune cells, but also their access to the tumor parenchyma, where the efficacy of immunotherapies must be deployed [75]. To infiltrate a tumor, immune cells must enter the tumor’s blood vessels, adhere to the endothelium, and transmigrate through the vessel wall [76]. All this may be prevented by the presence of an aberrant tumor vasculature, which could also explain the establishment of the immune-excluded tumor phenotype, commonly identified in BC, and associated with anti-PD1 resistance [77]. In this regard, it is noteworthy that human ductal BCs are characterized by high levels of VEGF-C/D leading to the decreased expression of endothelial adhesion molecules such as ICAM-1. This lowers the adhesion, extravasation, and infiltration of leukocytes into the tumor bed, thus establishing a physical barrier for their intratumor trafficking [78,79].

Moreover, angiogenic factors detectable at high levels in the TME can induce tumor-associated immune suppression through several mechanisms. First, VEGF inhibits dendritic cell (DC) maturation and antigen presentation, thereby hindering T-cell activation and consequently reducing the T-cell-mediated antitumor immune response [80]. Second, increased levels of angiogenic factors correspond to a direct inhibition of cytotoxic T lymphocytes’ (CTLs’) trafficking, proliferation, and effector function [81,82]. Third, high amounts of pro-angiogenic messengers promote the intratumor recruitment and proliferation of immunosuppressive cells [83,84]. All these processes can simultaneously occur in BCs.

Terminally differentiated DCs are key players in adaptive antitumor immunity [85] and secrete cytokines including IL-12 and IL-18 that inhibit endothelial cell proliferation [86,87]. However, tumor cells release other cytokines (e.g., VEGF, β-defensin, CXCL12, HGF, and CXCL8) that recruit immature DCs from the peripheral blood in the TME and, at the same time, hamper DCs’ maturation and function [88]. In this regard, in vitro experiments have shown that BC-derived cell lines secrete VEGF, which, in turn, inhibits the differentiation, maturation, and function of DCs from the healthy donor, and that VEGF gene silencing is followed by an increase in the expression of activation markers such as CD80, CD83, CD86, and HLA-DR on the DC’s surface [89]. The VEGF inhibitory effect on DC maturation has also been confirmed in animal models of BC [90].

Notably, when it is overexpressed, VEGF-A directly interferes with hematopoiesis [91] and impairs T-cell development in the thymus [84]: both effects are likely to be involved in the immune compromission observed in BCs. The binding of VEGF-A to the VEGFR expressed by T cells also contributes to their exhaustion status [81,92], a phenomenon characterized by the co-expression of several ICs, such as PD-1, T-cell immunoglobulin mucin-3 (Tim-3), cytotoxic T-lymphocyte-associated protein (CTLA-4), and lymphocyte activation gene 3 (Lag3), which results in a gradual loss of lymphocyte effector function [93]. VEGF-A has been reported to increase the expression of PD-1, CTLA-4, Tim-3, and Lag3 on CD8+ T cells in a variety of tumors [81]. In BC tissues, the expression of VEGF-A is positively correlated to that of PD-L1 [94]. Of interest, high levels of VEGF-A and PD-L1 parallel a low number of TILs in the BC-TME [94]. Consistently, low levels of VEGF-A are accompanied by an abundance of CD8+ T cells in the TME of BCs, and this predicts a long disease-free survival in BC patients [95,96,97].

Finally, the tumor vasculature stimulates the function of protumor immune cells in BC [98]. Among them, M2-like protumor macrophages (TAM), regulatory T cells (Tregs), and myeloid-derived suppressor cells (MDSCs) play an important role in BC progression [99,100].

Based on the type of stimuli they are subjected to, TAMs polarize into classically activated macrophages (M1, characterized by the expression of antitumoral cytokines) or into alternatively activated macrophages (M2, thought to be involved in cancer progression) [101]. In BC, TAM polarization is regulated by several TME-derived factors [102]. For example, upon VEGF binding to VEGFR2 expressed on their membrane, TAMs polarize to an M2-like phenotype [103,104] and secrete angiogenic and immunosuppressive cytokines (e.g., IL-10, TGFβ, and VEGF) that favor tumor progression [105,106,107]. Interestingly, macrophages of BC-bearing mice have been found to express both VEGFR1 and VEGFR2, while those from tumor-free mice express only VEGFR1 [90]. Analogously, VEGFR2+/CD45bright/CD14+ monocytes are present in the blood of BC patients but not healthy controls [108]. Not surprisingly, a high number of tumor-infiltrating macrophages is a marker of poor prognosis for BC patients [102].

On their part, Tregs secrete VEGFA in a fashion paralleling BC clinical progression [109]. In this regard, one should consider that the Treg-specific transcription factor FOXP3 cooperates with STAT3 to induce VEGF-A expression in Tregs, thus triggering angiogenesis [109]. Since they positively correlate not only with VEGF expression and BC vascularity but also with BC growth rate, invasiveness, and metastasis, FOXP3 levels have been thought to be capable of monitoring BC clinical progression [110].

The immunosuppressive Tregs are recruited in the TME by the MDSCs that populate BCs [111]. Recently, it has been shown that BC cells release both IL-34, that induces myeloid stem cells’ differentiation into monocytic MDSCs [111], and CXCL17, that promotes the accumulation of MDSCs within the lung where they favor the development of a metastatic niche [112]. Results from further animal studies indicate that BC metastases to the lung are facilitated by the loss of the *Shb* gene in endothelial cells, which is followed by the recruitment of monocytic MDSCs in the lung [113]. Altogether, these findings explain why the infiltration of BC by MDSCs or an increase in MDSCs in peripheral blood correlate with BC progression and metastatic burden in patients [114].

Notably, the hypoxia and acidosis present in TME because of the abnormal tumor vasculature can in turn promote local and systemic immunosuppression. In immune competent syngeneic BC mouse models, factors secreted by hypoxic tumor cells recruit CD11b+/Ly6Cmed/Ly6G+ myeloid cells and suppress natural killer (NK) cell functions [115]. In BC cells, hypoxia induces the expression of BIRC2, which counters the capability of CXCL9 to recruit CD8+ T cells and NK cells to the tumor, and hence increases tumor growth and resistance to anti-PD-1 therapy [116]. In hypoxic BC cells, the HIF-1 transcription factor is activated together with anaerobic metabolism and lactate dehydrogenase (LDHA) production [117]. The expression of both HIF1α and LDH5 defines “cold”, immunologically silent BCs and poor prognosis of patients [117].

## 4. Vessel Normalization Strategies in Breast Cancer

It has long been known that drugs inhibiting the formation of new blood vessels or damaging already formed tumor vessels can delay cancer progression [118]. However, antiangiogenic agents, often used at high doses, have shown some limitations in clinical applications since the destruction of blood vessels caused by these drugs promotes hypoxia, which, as we have already reported, accelerates tumor progression [119].

To date, growing evidence indicates that normalization rather than destruction of the tumor vasculature might be an effective antitumor strategy. Vascular normalization involves the judicious dosing of antiangiogenic agents to reverse the abnormal phenotype of the tumor vasculature [120]. To this end, the restoration of structurally and functionally fit blood vessels will be achieved through a series of normalizing events that include the fostering of a tighter connection between adjacent endothelial cells, a greater pericyte coverage, and the restoration of vascular basement membrane integrity to decrease vascular permeability and interstitial fluid pressure [121]. Although the structure and function of tumor vessels are unlikely to become completely normal (hence the term “normalized vessels”), this reversion can transiently render the distribution of blood flow more uniform and reduce the area of anoxia and acidosis within tumors [122]. Thus, the direct and anticipated consequences of vessel normalization are: (1) a strengthened immune response against cancer cells, through both vessel maturation and the relief of immunosuppression induced by hypoxia and/or angiogenic factors; (2) improved delivery of anticancer therapeutics and oxygen into the tumor bed; and (3) a decreased likelihood by the tumor to metastasize [100].

### Vessel Normalization Improves Immunotherapy and Vice Versa: Preclinical Evidence in Breast Cancer

**Figure 2:**
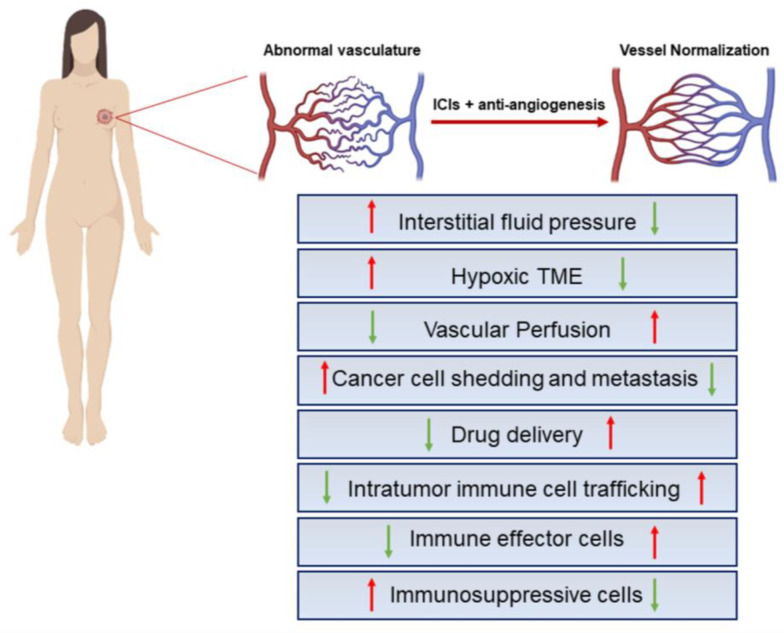
Key effects of IC blockers combined with antiangiogenics. Structural and functional abnormalities of tumor blood vessels lead to impaired blood flow and perfusion, hypoxic TME, limited drug delivery to the tumor, increased invasiveness of tumor cells, enhanced tumor infiltration of immunosuppressive cells, and impaired antitumor immune responses. ICI administration in combination with antiangiogenic factors may subvert this scenario in support of an immunosupportive BC-TME. The red and green arrows represent the indicated hyper-activated or down-modulated cellular processes, respectively. The figure was created with BioRender.com.

Recently, reciprocal interactions between the remodeling of tumor vessels and the reprogramming of the immune microenvironment have been demonstrated. On one side, in fact, vascular normalization enhances vascular perfusion and thereby increases intratumor infiltration of immune cells; on the other side, activated immune cells have a key role in normalizing the tumor vasculature [123,124,125]. For this reason, several preclinical studies have also been carried out in models of BC, showing that the inhibition of angiogenesis alone or in combination with various immunotherapies boosts antitumor immunity even in this aggressive neoplasm (Figure 2).

For instance, the use of BC murine models has shown that targeting the tumor vasculature with low doses of anti-VEGFR2 antibodies not only results in a homogeneous distribution of functional tumor vessels, but also facilitates tumor infiltration by CD4^+^ and CD8^+^ T cells and reverts the TAM phenotype from the pro-tumorigenic M2-like one to the antitumor, M1-like one [126]. Moreover, administration of the VEGF inhibitor bevacizumab to BC-bearing mice reduces the intratumor infiltration of protumor TAMs and MDSCs, as well as diminishing tumor vessel density and BC growth [90]. Similarly, mice treatment with DC101 (a rat monoclonal antibody directed against mouse VEGFR2), in addition to suppressing BC growth, attenuates the MDSCs’ inhibitory effect on T cells and reduces the number of Tregs in both primary BCs and lung metastases of BC [127]. Likewise, VEGF165b, an antiangiogenic isoform of VEGF-A, inhibits the MDSCs’ and Tregs’ accumulation in the spleens and tumors of BC-bearing mice [128].

In recent years, the possibility of combining antiangiogenic drugs with immunotherapy has grown to achieve an even better clinical outcome than that provided by the individual approaches. Specifically, consistent with the fact that BCs overexpressing the *Neu* proto-oncogene display high VEGF levels, the combination of DC101 with Neu-specific vaccination accelerates tumor regression in murine models of BC by augmenting the cytotoxic activity of CD8+ T cells [129].

Similar effects have been obtained in BC-bearing mice treated with the immune-stimulator recombinant fusion protein B7.2-IgG in combination with SU6668an, an inhibitor of the tyrosine kinase activity of three angiogenic receptors, namely VEGFR2, PDGFR-beta, and FGFR1 [130].

Furthermore, other studies have supported the rationale of co-targeting angiogenesis and ICIs for BC therapy, positioning immune cells as key effectors of antiangiogenic agents [131,132,133,134,135,136,137,138,139].

The combination of DC101 with anti-PD-L1 antibodies promotes the formation of high endothelial venules in mouse BC, enabling intratumor infiltration of cytotoxic T cells and thereby sensitizing BCs to anti-PD-L1 therapy [131].

In addition, the dual blockade of angiopoietin-2 and VEGF-A by a bispecific antibody (A2V) causes the normalization or regression of tumor vessels, the extravasation and perivascular accumulation of activated CD8+ cytotoxic T lymphocytes, the necrosis of BC, and, consequently, the presentation of neoantigens by intratumor phagocytes [132]. The concomitant blockade of PD-1 further enhances tumor control by A2V [132]. Similarly, a combination of angiopoietin-2 blockers, VEGF inhibitors, and anti-PD-L1 antibodies can successfully treat human metastatic BC xenografts and syngeneic BCs in mice [133].

More recently, Li et al. tested the combination of anti-PD-1 antibodies and different doses of VEGFR2-targeting agents in syngeneic BC mouse models, demonstrating a dose-dependent synergism between antiangiogenic therapy and IC blockade. Specifically, mice treatment with low doses of anti-VEGFR2 antibodies not only normalizes tumor vessels but also results in more robust immune cell infiltration, thus providing important insights into the optimal strategies for combining immunotherapy with molecular-targeted agents [134]. In the BC EMT-6/CDDP model, the administration of anti-PD-L1 antibodies is effective as an adjuvant monotherapy, while the combination of anti-PD-L1 antibodies with paclitaxel and VEGF antagonists gives better efficiency results in a neoadjuvant setting [135].

Notably, the use of several mouse models deficient in vascular normalization or T lymphocytes has allowed the delineation of an unexpected role of CD4+ T cells as a major immune cell population associated with vascular reprogramming. Indeed, the depletion of CD4+ T lymphocytes impairs vascular normalization [136]. In addition, ICIs facilitate vessel normalization in BC through a mechanism mediated by CD4+ T cells in an IFN gamma-dependent manner [136].

On the other hand, Zheng et al. primarily ascribed to CD8+ T cells’ activation the mechanism by which to achieve the increased vessel perfusion and antitumor effects of IC blockade. The authors demonstrated on several clinically relevant BC models, including orthotopic BCs (EO771, 4T1, and MCaP0008) and spontaneous BCs (MMTV-PyVT, which mirrors BC progression in humans), that CTLA4 and PD-1 antagonists increase blood perfusion of the tumor while they exert antitumor activities [123].

Intriguingly, Kabir et al. identified Myct1 as a new critical factor for tumor angiogenesis, nearly exclusively expressed in endothelial cells. The authors found that Myct1 expression is crucial for cancer progression through the regulation of both tumor angiogenesis and tumor immunity. Indeed, Myct1 deficiency reduces angiogenesis, and facilitates the trans-endothelial migration of cytotoxic T lymphocytes and the polarization of macrophages toward the M1 phenotype. Moreover, when Myct1 targeting is combined with anti-PD-1 treatment, the tumor regression is complete, with a significant long-term survival of BC-bearing mice [137]. Moreover, TNBC-bearing mice treated with the TGF-β inhibitor TRANILAST combined with the nanomedicine DOXIL as a vessel normalizing strategy, show a marked reduction in extracellular matrix components and an increase in intratumor vessel diameter and pericyte coverage: this leads to the infiltration of T cells and M1 macrophages into the tumor and improves the efficacy of anti-PD-1/anti-CTLA-4 antibodies [138]. Recently, nanocomplexes were prepared that release sunitinib (a vascular normalizing drug) and BMS-202 (a PD-1/PD-L1 blocker) in tumor tissues. The administration of such nanocomplexes to BC-bearing mice resulted in the significant inhibition of tumor growth coupled with excellent efficacy of antitumor immunity, which supports a potential new approach for BC treatment [139].

Importantly, all these studies suggest that in the case of combined therapeutic regimens, beyond identifying the suboptimal dose of the angiogenesis inhibitor, it is equally crucial to administer the other drugs, such as ICIs, during the window of normalization induced by the angiogenesis inhibitors. This will yield better results in terms of drug delivery into the tumor core as well as of triggering antitumor immunity.

## 5. Effect of Antiangiogenic Agents Combined with Immune Checkpoint Inhibitors in Breast Cancer: Clinical Studies 

Previous studies have evaluated the effect of bevacizumab added to neoadjuvant chemotherapy in TNBC patients. Despite an improvement in progression-free survival, this combination has resulted in an increased incidence of adverse events and has not augmented the overall survival of BC patients [140,141,142,143]. In general, the clinical benefit of antiangiogenic drugs as a monotherapy or in association with chemotherapy remains controversial in BC [144,145].

To date, based on the results from the previously illustrated preclinical studies providing evidence that angiogenesis-induced immunosuppression can be exploited to improve immunotherapy, the addition of antiangiogenic agents to ICIs is considered an attractive treatment approach. Hence, this novel combination therapy is currently being addressed in clinical trials for many malignances. Thus far, the Food and Drug Administration has approved combinations of ICIs and antiangiogenic agents for the treatment of renal cell carcinoma [146], non-small-cell lung cancer [147], hepatocellular carcinoma [148], and endometrial carcinoma [149]. Regarding BC, numerous phase I and II clinical trials are currently underway (Table 1).

An explorative analysis confirmed that (1) staining positivity for CD8 identifies TNBCs that are likely to benefit from immunotherapy, and (2) as angiogenesis discriminates patients with low CD8+ T-cell infiltration, angiogenesis inhibitors may facilitate IC blockade. These observations were preparatory for the launch of a phase II clinical trial (NCT04129996), which was conducted in 48 late-stage TNBC patients to assess the feasibility of combining FAMITINIB (an angiogenesis inhibitor), with CAMRELIZUMAB (a monoclonal antibody directed against PD-1) and conventional chemotherapeutics. Notably, patients’ response rate was above 80%, and no treatment-related deaths were reported [150].

Another phase II study (NCT04734262) evaluated the efficacy and safety of a chemotherapy-free regimen in which SITRAVATINIB (tyrosine kinase receptor inhibitor against the TYRO3, AXL, MERTK, and VEGF family) was given in combination with TISLELIZUMAB to patients with relapsed and/or metastatic TNBC, regardless of PD-L1 status. BC patients included in the study were divided into two cohorts: cohort A receiving 70 mg SITRAVATINIB plus 200 mg TISLELIZUMAB, and cohort B receiving 100 mg SITRAVATINIB plus 200 mg TISLELIZUMAB. Patients of cohort A demonstrated clinically significant antitumor activity and a manageable safety profile.

A recent phase II study (NCT04303741) employed CAMRELIZUMAB (an anti-PD-1 antibody) in combination with APATINIB (a tyrosine kinase inhibitor specifically directed against VEGFR2) and ERIBULIN (an inducer of tumor vessel normalization) [151]. This therapy resulted in the transformation of a “cold” tumor to an “inflamed” tumor and was demonstrated to be safe and effective in patients with heavily pretreated advanced TNBC, even in those negative for PD-L1, or in those who have progressed after several lines of treatment, including ICIs [151].

The phase II study NCT04914390 was initiated in August 2021 with the aim to evaluate the efficacy and safety of combining ANLOTINIB (a novel multitarget tyrosine kinase inhibitor that effectively inhibit VEGFR, FGFR, c-KIT, c-MET, and RET) with TISLELIZUMAB (a humanized immunoglobulin G4 anti-PD-1 monoclonal antibody engineered to minimize binding to FcγR on macrophages) and chemotherapy, as a neoadjuvant treatment in TNBC. The clinical trial will enroll a total of 32 patients, for which pCR rate, invasive disease-free survival, event-free survival, overall survival, and safety will be evaluated.

Additional studies are currently underway to evaluate the efficacy of this type of therapeutic combination in other BC subtypes. For instance, the pilot clinical trial NCT02802098 is seeking to explore the efficacy of combining the anti-PD-L1 durvalumab plus the antiangiogenic bevacizumab after bevacizumab monotherapy for advanced HER2-negative BC [152]. Peripheral blood mononuclear cells and fresh pre-durvalumab tumor biopsies have been analyzed to assess vascular normalization and to characterize the immune infiltrate. Preliminary results are encouraging and suggest that the antiangiogenic treatment exerts an immune-priming effect, with a systemic and intratumor reduction in Tregs [152].

In another recent phase Ib study (NCT02802098), patients with HER2-negative metastatic BC were treated with durvalumab plus bevacizumab. The results indicated that CD8+ memory effector T cells are increased in the peripheral blood from patients with stable BC but not from patients with progressed BC [153].

Recently, a patient affected by advanced metaplastic BC not responsive to standard adjuvant chemotherapy partially responded to the immunotherapeutic TORIPALIMAB combined with the angiogenesis inhibitor ANLOTINIB [154].

Finally, and most importantly, one should consider that as tumor vessel normalization ameliorates drug delivery into the tumor core, combination strategies require not only low doses of antiangiogenics but also even lower doses of ICIs: this improves antitumor immune responses, and simultaneously reduces the risk of clinical side effects that are associated with the administration of the individual therapeutic agents. Indeed, antiangiogenic therapy can suppress the reactive capillary hemangioma caused by anti-PD-1 antibodies [155,156] or reduce the risk of cerebral edema promoted by other ICIs [157], hence being effective in treating the brain metastases of BC [158].

## 6. Conclusions and Perspectives

Understanding the mechanisms of resistance to immunotherapy may enable the design of new therapeutic combinations that are hopefully more effective against BCs than the conventional ones.

The important role that tumor vessels play in evading the immune response is currently considered a major obstacle to overcome. Not surprisingly, clinical trials testing the combination of antiangiogenic agents with ICIs have increased since 2018 [75]. Nevertheless, efforts are needed to select the BC patients most likely to benefit from this therapeutic combination. Some studies have suggested that due to the transient window of antiangiogenic therapy and the low PD-L1 positivity rate in patients with advanced BC, an antiangiogenic therapy combined with immunotherapy may yield better clinical benefits in early-stage BC [159]. In contrast, results from other studies indicate that such a combined therapy is likely to be more promising in the neoadjuvant setting [159]. Therefore, to achieve more effective anti-BC therapies, the crosstalk between tumor vessels and immune cells has to be comprised, and many unsolved questions have to be answered. For instance, current combinatorial treatments focus on monoclonal antibodies as bevacizumab among antiangiogenics. However, the efficacy of this therapeutic approach is compromised over time by the induction of the expression of other angiogenic factors such as the FGFs [160]. Further studies will thus be needed to select additional antiangiogenic drugs in combination with the immunotherapy of choice. This could substantially improve the clinical outcomes of BC patients. In this regard, the effectiveness of combined antiangiogenics and immunotherapeutic against BC could be enhanced by the addition of chemotherapy and radiotherapy, which have been proven to increase the therapeutic efficacy of ICIs [161,162].

Further investigation should address the use of multimodal therapies directed against BC: timing, dosing, and toxicity will require careful consideration.

Finally, no reliable biomarkers predicting BC responsiveness to antiangiogenic agents or ICIs are available at the present time, and the selection of combination therapy is based on the positive results of monotherapies. Therefore, the identification of biomarkers foreseeing combination therapy outcomes would be of great importance.

## Figures and Tables

**Table 1:** Active, recruiting, and completed clinical studies of angiogenic inhibitors combined with ICIs for BC (data source: clinicalTrials.gov, December 2022).

Rank	NCT Number	Status	Conditions	Interventions
1	NCT03961698	Active, not recruiting	Breast Cancer, Renal Cell Carcinoma	IPI-549 (eganelisib), Atezolizumab, nab-paclitaxel, Bevacizumab
2	NCT03395899	Active, not recruiting	Breast Cancer, Estrogen Receptor-positive Breast Cancer	Atezolizumab, Cobimetinib, Ipatasertib, Bevacizumab
3	NCT03280563	Active, not recruiting	Breast Neoplasms	Atezolizumab (MPDL3280A), an engineered anti-programmed death-ligand 1 (PD-L1) antibody, Bevacizumab, Entinostat, Exemestane, Fulvestrant, Ipatasertib, Tamoxifen, Abemaciclib
4	NCT03387085	Active, not recruiting	Triple-Negative Breast Cancer	Aldoxorubicin HCl, N-803, ETBX-011, ETBX-051, ETBX-061, GI-4000, GI-6207, GI-6301, haNK for Infusion, avelumab, bevacizumab, Capecitabine, Cisplatin, Cyclophosphamide, 5-Fluorouracil, Leucovorin, nab-Paclitaxel, SBRT
5	NCT02734004	Active, not recruiting	Ovarian, Breast, SCLC, Gastric Cancers	Olaparib, MEDI4736, Bevacizumab
6	NCT03761914	Active, not recruiting	Breast Cancer, Other Tumors	galinpepimut-S, Pembrolizumab
7	NCT03170960	Active, not recruiting	Triple-Negative Breast Neoplasm, Other Solid Tumors	cabozantinib, atezolizumab
8	NCT02009449	Active, not recruiting	Solid Tumors, Breast Cancer	Pegilodecakin, Paclitaxel or Docetaxel and Carboplatin or Cisplatin, FOLFOX (Oxaliplatin/Leucovorin/5-Fluorouracil), gemcitabine/nab-paclitaxel, Capecitabine, Pazopanib, Pembrolizumab, Paclitaxel, nivolumab, Gemcitabine/carboplatin
9	NCT05431582	Not yet recruiting	Ovarian Cancer, Breast Cancer, Lung Cancer, Pancreatic Cancer	ZN-c3, Bevacizumab, Pembrolizumab
10	NCT04739670	Recruiting	Metastatic Triple-Negative Breast Cancer	Atezolizumab, Bevacizumab, Gemcitabine, Carboplatin
11	NCT04732598	Recruiting	Breast Cancer	Paclitaxel + bevacizumab therapy, Paclitaxel + bevacizumab + atezolizumab
12	NCT04408118	Recruiting	Metastatic Breast Cancer, Advanced Breast Cancer, Triple-Negative Breast Cancer	Atezolizumab, Paclitaxel, Bevacizumab
13	NCT05180006	Recruiting	Breast Cancer	Atezolizumab Injection, Ipatasertib, Bevacizumab, Pertuzumab, Trastuzumab
14	NCT05007106	Recruiting	Triple-Negative Breast Neoplasm, Other Solid Tumors	Pembrolizumab/Vibostolimab Co-Formulation, Pembrolizumab, Lenvatinib, 5-Fluorouracil, Cisplatin, Paclitaxel, Gemcitabine, Carboplatin, Docetaxel, Bevacizumab, Capecitabine, Oxaliplatin
15	NCT05092373	Recruiting	Advanced Breast Carcinoma, Advanced Other Solid Tumors	Atezolizumab, Cabozantinib S-malate, Nab-paclitaxel, Tumor Treating Fields Therapy
16	NCT04514484	Recruiting	Advanced Triple-Negative Breast Carcinoma, Other Advanced Solid Tumors	Cabozantinib S-malate, Computed Tomography, Magnetic Resonance Imaging, Nivolumab
17	NCT04802759	Recruiting	Inoperable, Locally Advanced or Metastatic, ER-positive Breast Cancer	Giredestrant, Abemaciclib, Ipatasertib, Inavolisib, Ribociclib, Everolimus, Samuraciclib, PH FDC SC, Palbociclib, Atezolizumab
18	NCT04591431	Recruiting	Breast Cancer, Gastrointestinal Cancer, Non-Small-Cell Lung Cancer	Erlotinib, Trastuzumab, Trastuzumab emtansine, Pertuzumab, Lapatinib, Everolimus, Vemurafenib, Cobimetinib, Alectinib, Brigatinib, Palbociclib, Ponatinib, Vismogedib, Itacitinib, Ipatasertib, Entrectinib, Atezolizumab, Nivolumab, Ipilimumab, Pemigatinib, Oncology Drugs
19	NCT03878524	Recruiting	Anatomic Stage IV Breast Cancer AJCC v8, Metastatic Breast Carcinoma, Other Advanced Solid Tumors	Abemaciclib, Abiraterone, Afatinib, Bevacizumab, Bicalutamide, Biospecimen Collection, Bortezomib, Cabazitaxel, Cabozantinib, Capecitabine, Carboplatin, Celecoxib, Cobimetinib, Copanlisib, Dabrafenib, Dacomitinib, Darolutamide, Dasatinib, Doxorubicin, Durvalumab, Enasidenib, Entrectinib, Enzalutamide, Erlotinib, Everolimus, Fluorouracil, Idelalisib, Imatinib, Ipilimumab, Lenvatinib, Leucovorin, Lorlatinib, Losartan, Nab-paclitaxel, Neratinib, Nivolumab, Olaparib, Oxaliplatin, Palbociclib, Panobinostat, Pembrolizumab, Pertuzumab, Ponatinib, Quality-of-Life Assessment, Regorafenib, Ruxolitinib, Sirolimus, Sorafenib, Sunitinib, Trametinib, Trastuzumab Emtansine, Tretinoin, Vemurafenib, Venetoclax, Vismodegib, Vorinostat
20	NCT02802098	Completed	Metastatic Breast Cancer, Bevacizumab-alone Maintenance Treatment Progression	Durvalumab, Bevacizumab
21	NCT03316586	Completed	Breast Cancer	Nivolumab, Cabozantinib

## Data Availability

Not applicable.

## Abbreviations

BC	breast cancer
CTL	cytotoxic T lymphocytes
CTLA-4	cytotoxic T-lymphocyte-associated protein
DC	dendritic cell
FGF	fibroblast growth factor
HER2	human epidermal growth factor receptor 2
HIF	hypoxia-inducible transcription factor
IC	immune checkpoint
ICIs	immune checkpoint inhibitors
IL-8	interleukin 8
Lag3	lymphocyte activation gene 3
LDHA	lactate dehydrogenase
MDSCs	myeloid-derived suppressor cells
MMPs	matrix metalloproteinases
pCR	pathologic complete response
PDGF	platelet-derived growth factor
PD-L1	programmed death-ligand 1
TAM	M2-like protumor macrophages
TGFβ-1	transforming growth factor beta-1
TILs	tumor-infiltrating lymphocytes
Tim-3	T-cell immunoglobulin mucin-3
TME	tumor microenvironment
TNBCs	triple-negative BCs
Tregs	regulatory T cells
VEGF	vascular endothelial growth factor
